# Demographic Disparities in Diagnosis and Treatment of Anxiety and Depressive Disorders in Head and Neck Cancer Survivors

**DOI:** 10.1002/hed.28103

**Published:** 2025-02-10

**Authors:** Tyler J. Gallagher, Ryan S. Chung, Nyree Khachikyan, Matthew E. Lin, Niels C. Kokot

**Affiliations:** ^1^ Keck School of Medicine of the University of Southern California Los Angeles California USA; ^2^ Department of Head and Neck Surgery, David Geffen School of Medicine University of California Los Angeles Los Angeles California USA; ^3^ Keck School of Medicine of the University of Southern California Department of Otolaryngology‐Head & Neck Surgery Los Angeles California USA

**Keywords:** anxiety, depression, disparities, head and neck cancer

## Abstract

**Background:**

The associations between head and neck cancer (HNC) and anxiety and depression are well‐known, but knowledge regarding the association between demographic factors and rates of diagnosis and treatment for these disorders among HNC survivors is incomplete.

**Methods:**

This retrospective study utilized propensity score matching to compare rates of selected new anxiety and/or depressive disorder diagnoses among HNC survivors and treatment of those disorders by sex, race, ethnicity, and language.

**Results:**

Results suggested lower likelihood of anxiety and/or depressive disorder diagnosis among individuals of male sex, and any minority, African American, Asian, and Other race, while diagnosis rates were higher among native Hawaiian/Pacific Islanders. Treatment with pharmacotherapy was less likely among individuals of male sex, any minority and African American Asian race, Hispanic ethnicity, and Spanish language.

**Conclusions:**

This study suggests that mental health diagnosis and treatment among HNC survivors may be inequitable, although further research is necessary to confirm these associations and understand underlying mechanisms.

## 1 Introduction

The association between head and neck cancer (HNC) and psychological issues, such as anxiety and depression, has been well‐documented in the literature [1, 2]. A variety of factors may contribute to development of anxiety or depressive disorders in this population, including fear of cancer progression, role changes, and diminished sense of control. Some may undergo potentially disfiguring HNC operations which can also contribute to the development of anxiety and depression, social isolation, and changes in relationships [1]. The surgical treatment of this malignancy, as well as any adjuvant chemoradiation, can lead to changes in patients' ability to breathe, speak, eat, and hear [3]. For these reasons, development of new psychosocial problems are very common in HNC survivors [4, 5].

Studies among other populations have shown that discrepancies still exist between racial and ethnic groups in the diagnosis of mental health disorders, referrals to counseling, and antidepressant therapy utilization [6, 7]. These discrepancies can be due to perpetuated stigma, clinician failures to engage patients, and poor ability on behalf of the patient to manage their own health [8]. Additionally, racial and ethnic minorities are often disproportionately affected by factors such as a limited income, language barriers, cultural biases, and incarceration [9]. These all lead to a higher rate of undiagnosed or misdiagnosed mental health disorders. Undiagnosed and misdiagnosed mental health problems may contribute to worse outcomes in cancer patients. Depression is associated with poor adherence to medical treatment; a study in a general population showed that compared to non‐depressed patients, depressed patients were three times as likely to be nonadherent with treatment recommendations from their physicians [10].

While discrepancies in the rate of diagnosis and treatment of mental health disorders among a general population have been documented previously in literature, it is unclear how race and ethnicity may specifically affect the diagnosis of anxiety and depression in HNC patients. In our retrospective study, we leveraged a large database of electronic medical record data to search for discrepancies in the diagnosis and treatment of new anxiety and depressive disorders among HNC survivors based on sex, race, ethnicity, and primary language. We hypothesized that psychological disorders are underdiagnosed and undertreated in minority HNC patients.

## 2 Materials and Methods

This study received exempt approval from the University of Southern California Institutional Review Board (UP‐23‐00652).

### 2.1 Study Population

This is a retrospective cohort study utilizing TriNetX, a globally federated health research network—a collection of secure health claims data—that provides access to consistently updated deidentified electronic medical record data [11]. We utilized the US Collaborative network, which provided access to data from 64 healthcare organizations (HCOs), primarily tertiary care centers, and accounted for over 110 million individuals. TriNetX LLC is compliant with the Health Insurance Portability and Accountability Act (HIPAA), as information is supplied only in aggregated counts of 10 or greater with deidentified information only.

Multiple sets of cohorts were created. All cohorts were required to have met criteria for inclusion from between November 22, 2004, to November 22, 2024. The first cohort was defined by diagnosis of a HNC without a prior diagnosis of a selected anxiety disorder (described in Section 2.2) or a depressive disorder. Individuals with other mental health conditions were not excluded, as many other mental health conditions are less directly associated with HNC. The second cohort was defined by diagnosis of a HNC without a prior diagnosis of an anxiety or depressive disorder but with one or both diagnosed within 2 years after HNC diagnosis. All cohorts included adults (≥ 18 years) only. Each of these two cohort sets had sub‐cohorts defined by demographic groups, including by race (White, Black or African American, Asian, Other Race, American Indian or Alaskan Native, or Native Hawaiian or Other Pacific Islander), ethnicity (Not Hispanic or Latino and Hispanic or Latino), and primary language (English or Spanish language). Additionally, to increase power in the racial comparison, a cohort of “any minority” (any non‐White race) was also created. Furthermore, cohorts divided by age (< 60 and ≥ 60 years), chemotherapy and radiation history, comorbidity history (≥ 1 or < 1 of myocardial infarction, heart failure, or cerebral infarction), and by tumor stage (Stages 1 and 2 vs. Stages 3 and 4) were created for a supplemental analysis.

### 2.2 Outcomes of Interest

Two separate outcomes of interest were analyzed. First, among cohorts with HNC and no previous diagnosis of selected anxiety and/or depressive disorders, rate of new diagnosis of certain anxiety and/or depressive disorders within 2 years after HNC diagnosis was the first outcome of interest. Mental health conditions considered as outcomes including generalized anxiety disorder, panic disorder, acute stress disorder, post‐traumatic stress disorder, adjustment disorder, and other reactions to severe stress and other mixed anxiety disorders (classified for analysis as anxiety disorders) and single and recurrent major depressive episodes and unspecified depressive episodes (classified for analysis as depressive disorders). The 2‐year timeframe was selected, as new anxiety or depression disorder diagnosis within this timeframe is more likely to be connected to HNC diagnosis and treatment. Second, among cohorts with new anxiety and/or depressive disorder diagnoses within 2 years after HNC diagnosis, the rate of treatment of anxiety and/or depressive disorder with pharmacotherapy or psychotherapy was analyzed. Relevant pharmacotherapies were selected based on current clinical practice guidelines [12, 13]. Anxiolytics (such as benzodiazepines) were not included in analysis, as they are less specific medications and are not primarily recommended for treatment of anxiety or depressive disorders. Relevant ICD‐10, CPT, SNOMED, TriNetX, and RxNorm codes are listed in Table S1.

### 2.3 Statistical Analysis

TriNetX advanced analytics were utilized for all analyses. Advanced analytics were first utilized to describe the rate of certain new anxiety and/or depressive disorder diagnosis within 2 years after HNC diagnosis in each sub‐cohort. A similar analysis was performed to analyze the rate of utilization of pharmacotherapy and psychotherapy for each group after diagnosis of anxiety and/or depressive disorder. Then, propensity score 1:1 matching (with greedy nearest neighbor matching, and a caliper distance of 0.1 pooled standard deviations of the logit of the propensity score) was used to create cohorts with matched baseline demographic and relevant medical characteristics potentially associated with anxiety and/or depressive disorders (including age, sex, race, ethnicity, chemotherapy status, radiation status, summary staging, and history of stroke, heart failure, and myocardial infarction) with the exception of the variable being manipulated between cohorts. Odds ratios were subsequently calculated by directly diving the odds of the event in one group by the odds of the same event in the comparison group after propensity score matching (similar to previous methods) [14, 15] to compare the rate of selected new anxiety and/or depressive disorder diagnosis between sub‐cohorts after HNC diagnosis. In cases where data were missing, the proportion of missing covariate data was matched between groups through propensity score matching. Additionally, the rate of utilization of pharmacotherapy and psychotherapy to treat selected diagnosed anxiety and/or depressive disorder after diagnosis was also compared. A limited secondary analysis was completed that excluded individuals with any missing demographic data (including age, race, ethnicity, and primary language).

## 3 Results

### 3.1 Cohort Characteristics

The database included 251 688 individuals with HNC. After excluding 58 216 individuals diagnosed with selected anxiety or depressive disorders before HNC, the cohort consisted of 193 472 adults. Of these, 6257 (3.3%) were diagnosed with a new anxiety or depressive disorder, including 3252 (1.7%) with a depressive disorder and 4260 (2.2%) with an anxiety disorder, within 2 years after new HNC diagnosis. The average age at time of HNC diagnosis was 62.8 years old (SD = 14.5). The cohort was predominantly male (70.5%), White (71.4%) and non‐Hispanic (65.4%), with English being the most common primary language (59.4%). Notably, data were missing for sex in 0.7% of individuals, race in 11.6%, ethnicity in 28.9%, primary language in 38.9%, and malignancy stage in 88.3%. Further characterization of the cohort is listed in Table 1.

**TABLE 1:** Demographic characteristics of cohort (*n* = 193 472).

Characteristic	*n* (%)
Age of HNC diagnosis (mean, SD)	62.8 (14.5)
Sex	
Male	136 467 (70.5)
Female	55 588 (28.7)
Unknown	1417 (0.7)
Race	
White	138 180 (71.4)
Unknown	22 418 (11.6)
Black or African American	17 960 (9.3)
Asian	6839 (3.5)
Other	6197 (3.2)
Native Hawaiian or Other Pacific Islander	1285 (0.7)
American Indian or Alaskan Native	593 (0.3)
Ethnicity	
Not Hispanic or Latino	126 468 (65.4)
Unknown ethnicity	55 830 (28.9)
Hispanic or Latino	11 174 (5.8)
Primary language	
English	114 935 (59.4)
Unknown or Non‐English/Spanish language	75 187 (38.9)
Spanish	3350 (1.7)
Malignancy summary stage	
Stage 1	5885 (3.0)
Stage 2	3854 (2.0)
Stage 3	4109 (2.1)
Stage 4	8717 (4.5)
Unknown	170 907 (88.3)
Comorbidity	
Acute myocardial infarction	8383 (4.3)
Heart failure	16 129 (8.3)
Cerebral infarction	8879 (4.6)

### 3.2 Differences in New Anxiety and Depressive Disorder Among HNC Survivors Based on Demographic Background

The percent of HNC survivors who received a new diagnosis of a selected anxiety and/or depressive disorder is summarized by demographic variables in Table 2. Grouped by category (sex, race, ethnicity, and primary language), the groups most diagnosed within 2 years after HNC diagnosis included females (4.76%), Native Hawaiian or Other Pacific Islanders (5.37%), Hispanic/Latinos (3.76%), and Spanish‐speakers (3.40%).

**TABLE 2:** Number and percent of HNC survivors receiving a selected anxiety and/or depressive disorder diagnosis^a^ within 2 years of HNC diagnosis by group.

Category	Group	Diagnosis	Total, *n*	Patients with outcome, *n* (%)
Sex	Male	Anxiety and/or depressive disorder	136 467	3615 (2.65)
	Female		55 588	2648 (4.76)
	Male	Anxiety disorder	136 467	2409 (1.77)
	Female		55 588	1859 (3.34)
	Male	Depressive disorder	136 467	1841 (1.35)
	Female		55 588	1411 (2.54)
Race	White/Caucasian	Anxiety and/or depressive disorder	138 180	4850 (3.51)
	Any minority		32 874	970 (2.95)
	Black or African American		17 960	588 (3.27)
	Asian		6839	122 (1.78)
	Other race		6197	171 (2.76)
	American Indian or Alaska Native		593	20 (3.37)
	Native Hawaiian or Other Pacific Islander		1285	69 (5.37)
	White/Caucasian	Anxiety disorder	138 180	3334 (2.41)
	Any minority		32 874	643 (1.96)
	Black or African American		17 960	371 (2.07)
	Asian		6839	81 (1.18)
	Other race		6197	123 (1.99)
	American Indian or Alaska Native		593	14 (2.36)
	Native Hawaiian or Other Pacific Islander		1285	54 (4.20)
	White/Caucasian	Depressive disorder	138 180	2520 (1.82)
	Any minority		32 874	499 (1.52)
	Black or African American		17 960	312 (1.74)
	Asian		6839	60 (0.88)
	Other race		6197	85 (1.37)
	American Indian or Alaska Native		593	11 (1.86)
	Native Hawaiian or Other Pacific Islander		1285	31 (2.41)
Ethnicity	Non‐Hispanic/Latino	Anxiety and/or depressive disorder	126 468	4206 (3.34)
	Hispanic/Latino		11 174	410 (3.76)
	Non‐Hispanic/Latino	Anxiety disorder	126 468	2847 (2.25)
	Hispanic/Latino		11 174	274 (2.45)
	Non‐Hispanic/Latino	Depressive disorder	126 468	2188 (1.73)
	Hispanic/Latino		11 174	239 (2.14)
Primary language	English	Anxiety and/or depressive disorder	114 935	3755 (3.27)
	Spanish		3350	114 (3.40)
	English	Anxiety disorder	114 935	2579 (2.24)
	Spanish		3350	73 (2.18)
	English	Depressive Disorder	114 935	1951 (1.70)
	Spanish		3350	65 (1.94)

^a^ Anxiety disorders included ICD‐10‐CM codes F41 and F43, while depressive disorders included ICD‐10‐CM codes F32 and F33.

Table S2 describes the relative rates of selected anxiety and/or depressive disorders based on age, history of chemotherapy, history of radiation, summary stage, and presence of comorbidities. Briefly, individuals age < 60 and those with a history of chemotherapy, radiation, and ≥ 1 comorbidity (myocardial or cerebral infarction or heart failure) had higher odds of new anxiety and/or depressive disorders. No difference in incidence of anxiety and/or depressive disorders was noted based on malignancy staging. Odds ratios were calculated directly from proportion in each group after propensity score matching to control for covariates.

Table 3 describes the odds ratios of receiving new anxiety and/or depressive disorder diagnosis, anxiety disorder diagnosis, and depressive disorder diagnosis within 2 years after HNC diagnosis by demographic variables after propensity score matching for all other demographic factors as well as chemotherapy and radiation status. Males were significantly less likely than females to receive these diagnoses (OR: 0.55 [95% CI: 0.52–0.59]). White individuals were more likely than a group of any minority (OR: 1.31 [95% CI: 1.20–1.43]), Asian individuals (OR: 1.94 [95% CI: 1.56–2.42]), and individuals of other races (OR: 1.39 [95% CI: 1.14–1.70]) to receive a selected diagnosis of anxiety and/or depressive disorder. Whites were also more likely to receive either or both diagnoses compared to Black individuals (OR: 1.28 [95% CI: 1.14–1.42]), though the association was not specific for depressive disorder alone. No significant difference was found between White individuals American Indians or Alaskan natives, while White individuals were less likely to receive diagnosis of anxiety and/or depressive disorders compared to Native Hawaiians or Other Pacific Islanders (OR: 0.63 [95% CI: 0.41–0.96]), though the association was not significant for depressive disorders alone. Additionally, no significant difference was noted between non‐Hispanics and Hispanics, or primary English speakers and Spanish language speakers.

**TABLE 3:** Odds ratios comparing likelihood of receiving diagnosis of a selected anxiety and/or depressive disorder^a^ within 2 years after HNC diagnosis based on demographic characteristics, after propensity matching for demographic and clinical variables.^b^

Group 1	Group 2	Diagnosis type	*n* per group after propensity score matching	Group 1: new diagnosis, *n* (%)	Group 2: new diagnosis, *n* (%)	OR [95% CI]	*p*
Male	Female	Anxiety and/or depressive disorder	55 601	1492 (2.68)	2617 (4.76)	0.55 [0.52–0.59]	< 0.001
		Anxiety disorder		992 (1.78)	1859 (3.34)	0.53 [0.49–0.57]	< 0.001
		Depressive disorder		752 (1.35)	1410 (2.54)	0.53 [0.48–0.58]	< 0.001
White/Caucasian	Any minority	Anxiety and/or depressive disorder	32 882	1260 (3.83)	970 (2.95)	1.31 [1.20–1.43]	< 0.001
		Anxiety disorder		889 (2.70)	643 (1.96)	1.39 [1.26–1.54]	< 0.001
		Depressive disorder		629 (1.91)	499 (1.52)	1.27 [1.12–1.43]	< 0.001
White/Caucasian	Black or African American	Anxiety and/or depressive disorder	17 813	739 (4.15)	585 (3.28)	1.28 [1.14–1.42]	< 0.001
		Anxiety disorder		526 (2.95)	370 (2.08)	1.43 [1.25–1.64]	< 0.001
		Depressive disorder		360 (2.02)	310 (1.74)	1.17 [0.99–1.36]	0.051
White/Caucasian	Asian	Anxiety and/or depressive disorder	6839	233 (3.41)	122 (1.78)	1.94 [1.56–2.42]	< 0.001
		Anxiety disorder		155 (2.27)	81 (1.18)	1.94 [1.48–2.54]	< 0.001
		Depressive disorder		121 (1.78)	60 (0.88)	2.04 [1.49–2.78]	< 0.001
White/Caucasian	Other race	Anxiety and/or depressive disorder	6189	234 (3.78)	170 (2.75)	1.39 [1.14–1.70]	0.001
		Anxiety disorder		160 (2.59)	122 (1.97)	1.32 [1.04–1.68]	0.022
		Depressive disorder		121 (2.00)	85 (1.37)	1.43 [1.08–1.89]	0.011
White/Caucasian	American Indian or Alaska Native	Anxiety and/or depressive disorder	593	20 (3.73)	20 (3.73)	1.00 [0.53–1.88]	0.999
		Anxiety disorder		14 (2.36)	14 (2.36)	1.00 [0.47–2.12]	0.999
		Depressive disorder		≤ 10 (≤ 1.69)	11 (1.86)	NA^c^	NA^c^
White/Caucasian	Native Hawaiian or oher Pacific Islander	Anxiety and/or depressive disorder	1138	35 (3.08)	55 (4.83)	0.63 [0.41–0.96]	0.032
		Anxiety disorder		25 (2.20)	44 (3.87)	0.56 [0.34–0.92]	0.020
		Depressive disorder		22 (1.93)	22 (1.93)	1.00 [0.55–1.82]	0.999
Non‐Hispanic/Latino	Hispanic/Latino	Anxiety and/or depressive disorder	10 977	389 (3.54)	419 (3.82)	0.93 [0.80–1.07]	0.282
		Anxiety disorder		267 (2.43)	274 (2.50)	0.97 [0.82–1.16]	0.761
		Depressive disorder		213 (1.94)	238 (2.17)	0.89 [0.74–1.08]	0.234
English primary language	Spanish primary language	Anxiety and/or depressive disorder	3306	135 (4.08)	108 (3.27)	1.26 [0.97–1.63]	0.078
		Anxiety disorder		82 (2.48)	70 (2.12)	1.18 [0.85–1.62]	0.325
		Depressive disorder		79 (2.39)	61 (1.85)	1.30 [0.93–1.83]	0.124

^a^ Anxiety disorders included ICD‐10‐CM codes F41 and F43, while depressive disorders included ICD‐10‐CM codes F32 and F33.

^b^ Odds ratios were calculated by directly diving the odds of the event in one group by the odds of the same event in the comparison group after propensity score matching for age, sex, race, ethnicity, English primary language, history of chemotherapy, history of radiation, tumor summary stage, and history of stroke, heart failure, or myocardial infarction. Race, ethnicity, and English primary language were removed from propensity score matching as necessary for comparisons.

^c^ TriNetX does not shown less than 10, so associated odds ratio calculations were omitted in these cases.

Table S3 describes the relative rates of selected anxiety and/or depressive disorders based on demographic factors with individuals with missing demographic data removed. Like the primary analysis, males, any minority, and Spanish speakers were less likely to receive a selected diagnosis of anxiety and/or depressive disorder, though this was not true specifically for depressive disorders only among minority individuals or anxiety disorders among Spanish speakers.

### 3.3 Differences in Treatment of Anxiety and Depression Based on Demographic Background

The percent of HNC survivors who received pharmacotherapy or psychotherapy after diagnosis of a selected anxiety and/or depressive disorder within first 2 years after HNC diagnosis, stratified by various sex, race, ethnicity, and primary language, is summarized in Table 4. Grouped by category, the groups most treated with pharmacotherapy (excluding groups with incomplete data due to small samples) include females (51.22%), White/Caucasians (49.99%), non‐Hispanic/Latinos (50.27%), and primary English speakers (49.50%). The groups most treated with psychotherapy include females (3.54%), Black/African Americans (4.36%), Hispanic/Latinos (5.38%), and primary English speakers (3.74%).

**TABLE 4:** Number and percent of HNC Survivors diagnosed with a selected anxiety and/or depressive disorder receiving medication or psychotherapy treatment.

Category	Group	Treatment	Total, *n*	Patients with outcome, *n* (%)
Sex	Male	Pharmacotherapy	4544	2101 (46.34)
	Female		3077	1576 (51.22)
	Male	Psychotherapy	4544	126 (2.77)
	Female		3077	109 (3.54)
Race	White/Caucasian	Pharmacotherapy	5995	2997 (49.99)
	Any minority		1171	467 (39.88)
	Black or African American		711	295 (41.49)
	Asian		163	68 (41.72)
	Other race		189	88 (46.56)
	American Indian or Alaska Native		25	≤ 10 (≤ 40.00^a^)
	Native Hawaiian or Other Pacific Islander		83	≤ 10 (≤ 12.05^a^)
	White/Caucasian	Psychotherapy	5995	177 (2.95)
	Any minority		1171	40 (3.42)
	Black or African American		711	31 (4.36)
	Asian		163	≤ 10 (6.14^a^)
	Other race		189	≤ 10 (≤ 5.29^a^)
	American Indian or Alaska Native		25	0 (0.00)
	Native Hawaiian or Other Pacific Islander		83	0 (0.00)
Ethnicity	Non‐Hispanic/Latino	Pharmacotherapy	5260	2639 (50.27)
	Hispanic/Latino		483	206 (42.65)
	Non‐Hispanic/Latino	Psychotherapy	5260	186 (3.54)
	Hispanic/Latino		483	26 (5.38)
Primary language	English	Pharmacotherapy	4578	2266 (49.50)
	Spanish		126	35 (27.78)
	English	Psychotherapy	4578	171 (3.74)
	Spanish		126	≤ 10 (≤ 7.94^a^)

^a^ TriNetX does not shown less than 10, so associated percent may be inflated due to small sample size.

Table 5 describes the odds ratios of receiving pharmacotherapy or psychotherapy after diagnosis of certain anxiety and/or depressive disorders with odds ratios calculated directly from relative numbers treated after propensity score matching of demographic and medical characteristics. Males were less likely than females to receive pharmacotherapy for anxiety and/or depressive disorder (OR: 0.79 [95% CI: 0.71–0.89]). White individuals were more likely than a group of any minority (OR: 1.51 [95% CI: 1.28–1.78]) and Black individuals (OR: 1.61 [95% CI: 1.30–1.99]) to receive pharmacotherapy. Analyses comparing White individuals to Asians, other races, American Indian or Alaska natives and Native Hawaiians or Other Pacific Islanders were not significant. Non‐Hispanic/Latinos were more likely to receive pharmacotherapy than Hispanic/Latinos (OR: 1.77 [95% CI: 1.09–2.87]). Primarily English speakers were more likely to receive pharmacotherapy than primary Spanish speakers (OR: 1.94 [95% CI: 1.10–3.42]). No significant differences were observed in the relative utilization of psychotherapy.

**TABLE 5:** Odds ratios comparing likelihood of receiving pharmacotherapy or psychotherapy after selected anxiety and/or depressive disorder within 2 years after HNC diagnosis based on demographic characteristics, after propensity matching for demographic and clinical variables.^a^

Group 1	Group 2	Treatment type	*n* per group after propensity score matching	*n* (%) treated, Group 1	*n* (%) treated, Group 2	OR [95% CI]	*p*
Male	Female	Pharmacotherapy	2480	1115 (45.00)	1259 (50.77)	0.79 [0.71–0.89]	< 0.001
		Psychotherapy		77 (3.11)	95 (3.83)	0.80 [0.59–1.09]	0.162
White/Caucasian	Any minority	Pharmacotherapy	1167	583 (49.96)	465 (39.85)	1.51 [1.28–1.78]	< 0.001
		Psychotherapy		38 (3.26)	29 (3.34)	0.94 [0.62–1.53]	0.908
White/Caucasian	Black or African American	Pharmacotherapy	704	375 (53.27)	292 (41.48)	1.61 [1.30–1.99]	< 0.001
		Psychotherapy		31 (4.40)	30 (4.26)	1.04 [0.62–1.73]	0.896
White/Caucasian	Asian	Pharmacotherapy	162	77 (47.53)	67 (41.36)	1.28 [0.83–1.99]	0.264
		Psychotherapy		≤ 10 (≤ 6.17)	≤ 10 (≤ 6.17)	NA^b^	NA^b^
White/Caucasian	Other race	Pharmacotherapy	187	80 (42.78)	88 (47.06)	0.84 [0.56–1.27]	0.406
		Psychotherapy		≤ 10 (≤ 5.35)	≤ 10 (≤ 5.35)	NA^b^	NA^b^
White/Caucasian	American Indian or Alaska Native	Pharmacotherapy	24	≤ 10 (≤ 41.67)	≤ 10 (≤ 41.67)	NA^b^	NA^b^
		Psychotherapy		0 (0.00)	0 (0.00)	NA^b^	NA^b^
White/Caucasian	Native Hawaiian or Other Pacific Islander	Pharmacotherapy	59	24 (40.67)	≤ 10 (≤ 16.95)	3.36 [1.43–7.91]^b^	0.004^b^
		Psychotherapy		≤ 10 (≤ 16.95)	0 (0.00)	NA^b^	NA^b^
Non‐Hispanic/Latino	Hispanic/Latino	Pharmacotherapy	134	76 (56.72)	57 (42.54)	1.77 [1.09–2.87]	0.020
		Psychotherapy		≤ 10 (≤ 7.46)	≤ 10 (≤ 7.46)	NA^b^	NA^b^
English primary language	Spanish primary language	Pharmacotherapy	103	48 (46.60)	32 (31.07)	1.94 [1.10–3.42]	< 0.001
		Psychotherapy		≤ 10 (≤ 9.71)	≤ 10 (≤ 9.71)	NA^b^	NA^b^

^a^ Odds ratios were calculated by directly diving the odds of the event in one group by the odds of the same event in the comparison group after propensity score matching for age, sex, race, ethnicity, English primary language, history of chemotherapy, history of radiation, tumor summary stage, and history of stroke, heart failure, or myocardial infarction. Race, ethnicity, and English primary language were removed from propensity score matching as necessary for comparisons.

^b^ TriNetX does not shown less than 10, so associated odds ratio calculations were omitted in these cases.

Table S4 describes the relative odds of treatment of selected anxiety and/or depressive disorders based on demographic factors with individuals with missing demographic data removed after propensity score matching. Similar to the primary analysis, males, any minority, non‐Hispanic/Latinos, and Spanish speakers were less likely to receive pharmacotherapy, but not psychotherapy, for selected anxiety and/or depressive disorders. Again, no significant differences were observed in the relative utilization of psychotherapy.

## 4 Discussion

In this retrospective study, we analyzed the incidence of selected new anxiety and/or depressive disorder diagnoses—as well as the treatment of such diagnoses—among patients with HNC when stratified by a variety of demographic variables, including sex, race, ethnicity, and primary language. After propensity score matching for selected demographic factors and treatment status, we found the following groups to be more likely to receive one or both of a certain anxiety and/or depressive disorder diagnosis than the comparison group: female more than male, White more than any minority, Black, Asian, and native Hawaiian or Other Pacific Islander. When comparing treatment among those diagnosed with anxiety and/or depressive disorder, we did not find a difference in the utilization of psychotherapy. However, we did find the following groups to be more likely to receive pharmacotherapy for anxiety or depressive disorder than their counterparts: female more than male, White more than any minority or Black individual, non‐Hispanic more than Hispanic, and English‐speaking more than Spanish‐speaking. Native Hawaiian/Pacific Islanders were more likely to receive diagnosis of at least one of a certain anxiety and/or depressive disorder than White individuals. These significant differences may represent disparities in treatment between these groups which may negatively impact patients' quality of life as well as the treatment of HNC and its sequelae.

Previous studies have demonstrated the relatively high prevalence of mental health disorders and psychological distress among individuals with HNC [16, 17, 18, 19], although knowledge of demographic correlates of mental health disorder diagnosis and treatment has been somewhat limited. A prior study found higher reports of mental health disorders among females with HNC compared to their male counterparts, as well as among individuals with Medicare as opposed to other types of health insurance, though this study was based on self‐report [20]. Other factors suggested to be associated with higher distress and mental health difficulties among HNC patients include advanced cancer stage, living alone, higher neighborhood deprivation scores, and higher posttreatment symptom burden [18, 21, 22]. While data have shown that minority groups were less likely to receive therapy for anxiety and depression than White patients in primary care and psychiatric settings [6, 7], our study is the first to our knowledge to suggest this disparity among HNC patients.

While demonstration of the association of race, ethnicity, and primary language with anxiety and depressive disorder diagnosis and treatment is important, identifying the causes of these associations is the next step in addressing this potential disparity. Knowledge of these contributors may help guide amelioration of suggested differences in diagnosis and treatment rates. First, it is important to acknowledge that while it is theoretically possible that minority patients experience less anxiety and depression after HNC diagnosis, this is an unlikely cause of the difference in diagnosis rates observed. A few previous studies have evaluated psychological distress among HNC survivors with prospectively collected survey‐based data with findings generally showing either similar rates of anxiety and depression symptoms across racial groups or slightly higher rates among minority groups in the United States [19, 23]. Among cancer patients generally, data have supported the idea that minority individuals are more prone to post‐traumatic stress [24], suggesting our results utilizing coding data are more likely to reflect underdiagnosis among minority groups as opposed to lower rates of psychological distress. Previous studies have demonstrated that lower socioeconomic status is associated with anxiety and depression after HNC [22], suggesting that minority groups likely face more difficulties during HNC treatment that would make eventual anxiety and depressive disorders more likely.

Though potential causes were not explored in this study, there are several potential explanations for why minorities with HNC may be less likely to receive diagnosis and subsequent treatment for mental health disorders. It is important to acknowledge that cultural factors and historical biases may influence treatment‐seeking among minority populations, as previously described among Black and Asian American cohorts without HNC [25, 26, 27]. Stigma placed on individuals diagnosed and treated for mental health disorders can be a significant cultural barrier to treatment. While overcoming historical biases and perceptions is challenging, it may be easier to overcome provider biases through sensitivity training, as provider biases have been proposed as another contributor to disparities in diagnosis and treatment of anxiety and depression in other populations [28, 29]. Finally, economic and insurance barriers likely play a role in ability to access mental health resources among HNC patients, particularly given the significant time and cost burden of HNC diagnosis and treatment itself [20, 30, 31].

While these and other challenges to achieving equitable mental health diagnosis and treatment for HNC survivors exist, there are several strategies that might help to decrease some of these potential differences. We suggest introducing standardized screening surveys, such as the Patient Health Questionnaire 9 and Generalized Anxiety Disorder 7 (translated and validated in other languages, as necessary), into the clinical workflow of HNC survivor's appointments for early identification of these common conditions for HNC patients. Additionally, it is critical to have a collection of treatment resources available to individuals from a variety of backgrounds and economic situations to support HNC survivors who express symptoms of distress.

Our findings have notable limitations. This is a retrospective study, so findings are preliminary and should be replicated in other samples. While we were able to summarize many details of our cohort, a significant amount of data was missing, particularly regarding malignancy summary stage. Completion of a secondary analysis of individuals without missing demographic data had a similar result, though this limited findings regarding smaller racial groups. Additionally, some details of the cohort that would be useful to include in analysis as covariates, including insurance status, income, recurrence status, current treatment status (i.e., active vs. completed), number of treatment modalities, and receipt of curative versus palliative care, were unavailable and may have influenced findings. To a significant extent, many of the differences noted in treatment of anxiety and/or depressive disorders are likely linked to ability to pay for treatment options. Our sample consisted of patients primarily from tertiary care centers, where private insurance is more likely, so findings cannot be generalized to all populations. In some cases, small sample size limited findings, particularly, for smaller racial groups in the United States. Potential inconsistency of diagnosis and reliance on medical record codes is another limitation. This study only studies a specific set of anxiety disorders (including generalized anxiety disorder, panic disorder, acute stress disorder, post‐traumatic stress disorder, adjustment disorder, and other reactions to severe stress and other mixed anxiety disorders) and depressive disorders (including single and recurrent major depressive episodes and unspecified depressive episodes). It is important to acknowledge that rates of anxiety and depressive disorders are likely underreported here, as it is well‐recognized that oncology clinicians often underdiagnose mental health conditions due to a focus on treatment of malignancy [32, 33]. Finally, outside referral for treatment of anxiety and/or depression is another likely limitation of our study, particularly, for psychotherapy, which may not be offered in many HCOs and therefore would not be included in our study findings.

Despite these limitations, the likely association between differences in demographic characteristics and diagnosis and treatment of mental health disorders has significant public health implications. Given the relationship between mental health status and treatment compliance noted in other populations [10, 34], the racial differences between diagnosis and treatment of anxiety and depression suggested in this manuscript may contribute to discrepancies in overall outcomes for various demographic groups in the United States [35, 36, 37]. In fact, depression has previously been associated with higher mortality in non‐HNC cancer patients [38, 39], further demonstrating the need for further research to explore any possible effects of anxiety and depression screening and intervention on disease outcomes [40]. Future studies should seek to prospectively replicate the findings of this manuscript while utilizing controls for income, education, and payor status. Additionally, future work could aim to gain understanding of the relative contribution of various factors—including culture and implicit bias—to these potential disparities in order to address them in the future.

## 5 Conclusion

This cohort study suggests an association between several demographic factors and likelihood of receiving a certain anxiety or depressive disorder diagnosis after HNC diagnosis. Additionally, this study suggests similar associations between a variety of demographic factors and likelihood of receiving treatment for new mental health disorders after HNC diagnosis. Further research is necessary to investigate these relationships and better meet the unique healthcare needs of patients diagnosed with head and neck cancer.

## Author Contributions

Interpretation of data, drafting of manuscript, critical revision, and final approval of the submission: Tyler J. Gallagher. Data acquisition and analysis, interpretation of data, drafting of manuscript, critical revision: Ryan S. Chung, Nyree Kachikyan, and Matthew E. Lin. Study conception, study design, data acquisition and analysis, interpretation of data, drafting of manuscript, critical revision, and final approval of the submission: Niels C. Kokot.

## Conflicts of Interest

The authors declare no conflicts of interest.

## Supporting information


**Data S1.** Supporting Information.

## Data Availability

The data that support the findings of this study are available from TriNetX. Restrictions apply to the availability of these data, which were used under license for this study. Data are available from https://trinetx.com/ with the permission of TriNetX.
