# Supplementary material for: Demographic Disparities in Diagnosis and Treatment of Anxiety and Depressive Disorders in Head and Neck Cancer Survivors
Source: Head Neck. 2025 Feb 10;47(7):1885–96. doi: 10.1002/hed.28103 (PMC12146807; doi:10.1002/hed.28103)
Supplement: Supplementary file 1 — Data S1. Supporting Information. [file HED-47-1885-s001.docx]

**Supplemental Table 1.** Relevant ICD-10, CPT, SNOMED, TriNetX, and RxNorm Codes

| **Code Type** | **Code** | **Name** | **Grouping(s) for Analysis** |
| --- | --- | --- | --- |
| ICD-10-CM | C00-C14, C30-32 | Malignant neoplasms of lip, oral cavity, pharynx, nasal cavity, larynx | Head and Neck Cancers |
|  | F32 | Depressive episode (including single major or unspecified depressive episodes) | Depressive disorder |
|  | F33 | Major depressive disorder, recurrent | Depressive disorder |
|  | F41 | Other anxiety disorders (including generalized anxiety disorder, panic disorder, and other specified and unspecified anxiety disorders) | Anxiety disorder |
|  | F43 | Reaction to severe stress (includes acute stress reactions, reactions to severe stress, other reactions to severe stress, and other mixed anxiety disorders) | Anxiety disorder |
|  | I21 | Acute myocardial infarction | Myocardial infarction (control) |
|  | I50 | Heart failure | Heart failure (control) |
|  | I63 | Cerebral infarction | Stroke (control) |
| ICD-10-PCS | GZH | Group Psychotherapy | Psychotherapy |
|  | GZ5 | Individual Psychotherapy |  |
| CPT | 90847 | Family Psychotherapy |  |
|  | 90853 | Group Psychotherapy |  |
|  | 90832 | Psychotherapy, 30 minutes |  |
|  | 90833 | Psychotherapy, 30 minutes (evaluation and management) |  |
|  | 90834 | Psychotherapy, 45 minutes |  |
|  | 90836 | Psychotherapy, 45 minutes (evaluation and management) |  |
|  | 90837 | Psychotherapy, 60 minutes |  |
|  | 90838 | Psychotherapy, 60 minutes (evaluation and management) |  |
| SNOMED | 75516001 | Psychotherapy |  |
| RxNorm | 15996 | Mirtazapine | Pharmacotherapy for Anxiety and/or Depression |
|  | 42347 | Bupropion |  |
|  | 4493 | Fluoxetine (SSRI) |  |
|  | 36437 | Sertraline (SSRI) |  |
|  | 32937 | Paroxetine (SSRI) |  |
|  | 42355 | Fluvoxamine (SSRI) |  |
|  | 2556 | Citalopram (SSRI) |  |
|  | 321988 | Escitalopram (SNRI) |  |
|  | 39786 | Venlafaxine (SSRI) |  |
|  | 734064 | Desvenlafaxine (SNRI) |  |
|  | 72625 | Duloxetine (SNRI) |  |
|  | 1455099 | Vortioxetine |  |
|  | 1086769 | Vilazodone |  |
| TNX Curated | 1002 | Chemotherapy | Chemotherapy (control) |
|  | 1001 | Radiation | Radiation (control) |

**Supplemental Table 2.** Odds ratios^a^ comparing likelihood of receiving diagnosis of a selected anxiety and/or depressive disorder within 2 years after HNC diagnosis^b^ based on other demographic and clinical variables, after propensity score matching

| **Group 1** | **Group 2** | **Diagnosis Type** | **n per group after propensity score matching** | **n (%) New Diagnosis, Group 1** | **n (%) New Diagnosis, Group 2** | **OR [95% CI]** | **p-value** |
| --- | --- | --- | --- | --- | --- | --- | --- |
| <60 years | ≥60 years | Anxiety and/or Depressive Disorder | 32,838 | 1,273 (3.88%) | 1,040 (3.17%) | **1.23 [1.13-1.34]** | **<0.001** |
|  |  | Anxiety Disorder |  | 931 (2.84%) | 709 (2.16%) | **1.32 [1.20-1.46]** | **<0.001** |
|  |  | Depressive Disorder |  | 648 (1.98%) | 559 (1.70%) | **1.16 [1.04-1.31]** | **<0.001** |
| Chemo- therapy | No Chemo- therapy | Anxiety and/or Depressive Disorder | 40,134 | 3,557 (8.86%) | 1,180 (2.94%) | **3.21 [3.00-3.43]** | **<0.001** |
|  |  | Anxiety Disorder |  | 2,528 (6.30%) | 804 (2.00%) | **3.29 [3.03-3.56]** | **<0.001** |
|  |  | Depressive Disorder |  | 1,784 (4.45%) | 605 (1.51%) | **3.04 [2.77-3.34]** | **<0.001** |
| Radiation | No Radiation | Anxiety and/or Depressive Disorder | 34,075 | 3,655 (10.73%) | 1,248 (3.66%) | **3.16 [2.96-3.38]** | **<0.001** |
|  |  | Anxiety Disorder |  | 2,670 (7.84%) | 823 (2.42%) | **3.44 [3.17-3.72]** | **<0.001** |
|  |  | Depressive Disorder |  | 1,874 (5.50%) | 666 (1.96%) | **2.92 [2.67-3.19]** | **<0.001** |
| Stage 3 or Stage 4 | Stage 1 or Stage 2 | Anxiety and/or Depressive Disorder | 9,685 | 260 (2.69%) | 262 (2.71%) | 0.99 [0.83-1.18] | 0.992 |
|  |  | Anxiety Disorder |  | 186 (1.92%) | 173 (1.79%) | 1.08 [0.87-1.33] | 0.489 |
|  |  | Depressive Disorder |  | 122 (1.26%) | 139 (1.44%) | 0.88 [0.69-1.12] | 0.289 |
| ≥1 Comor-bidity^c^ | No Comor-bidity | Anxiety and/or Depressive Disorder | 29,799 | 1,828 (6.13%) | 780 (2.62%) | **2.43 [2.32-2.65]** | **<0.001** |
|  |  | Anxiety Disorder |  | 1,201 (4.03%) | 516 (1.73%) | **2.83 [2.15-2.65]** | **<0.001** |
|  |  | Depressive Disorder |  | 1,029 (3.45%) | 402 (1.35%) | **2.62 [2.33-2.94]** | **<0.001** |

^a^Odds ratios were calculated by directly diving the odds of the event in one group by the odds of the same event in the comparison group after propensity score matching for age, sex, race, ethnicity, English primary language, history of chemotherapy, history of radiation, tumor summary stage, and history of stroke, heart failure, or myocardial infarction. Race, ethnicity, and English primary language were removed from propensity score matching as necessary for comparisons.

^b^Anxiety disorders included ICD-10-CM codes F41 and F43, while depressive disorders included ICD-10-CM codes F32 and F33

**^c^**Comorbidities included in this analysis include acute myocardial infarction, heart failure, and cerebral infarction.

**Supplemental Table 3.** Odds ratios comparing likelihood of receiving diagnosis of a selected anxiety and/or depressive disorder^a^ within 2 years after HNC diagnosis based on demographic characteristics after propensity matching: sensitivity analysis omitting participants with missing demographic data^b^

| **Group 1** | **Group 2** | **Diagnosis Type** | **n per group after propensity score matching** | **n (%) New Diagnosis, Group 1** | **n (%) New Diagnosis, Group 2** | **OR [95% CI]** | **p-value** |
| --- | --- | --- | --- | --- | --- | --- | --- |
| Male | Female | Anxiety and/or Depressive Disorder | 25,169 | 676 (2.69%) | 1,330 (5.28%) | **0.50 [0.45-0.54]** | **<0.001** |
|  |  | Anxiety Disorder |  | 446 (1.77%) | 918 (3.65%) | **0.48 [0.43-0.53]** | **<0.001** |
|  |  | Depressive Disorder |  | 364 (1.45%) | 735 (2.92%) | **0.49 [0.43-0.55]** | **<0.001** |
| White/ Caucasian | Any minority^c^ | Anxiety and/or Depressive Disorder | 12,933 | 512 (3.96%) | 425 (3.29%) | **1.21 [1.06-1.38]** | **0.004** |
|  |  | Anxiety Disorder |  | 356 (2.75%) | 254 (1.96%) | **1.41 [1.20-1.66]** | **<0.001** |
|  |  | Depressive Disorder |  | 277 (2.14%) | 244 (1.89%) | 1.14 [0.96-1.35] | 0.144 |
| Non- Hispanic/ Latino | Hispanic/ Latino | Anxiety and/or Depressive Disorder | 3,397 | 144 (4.24%) | 149 (4.39%) | 0.97 [0.76-1.22] | 0.765 |
|  |  | Anxiety Disorder |  | 96 (2.83%) | 95 (2.80%) | 1.01 [0.76-1.35] | 0.942 |
|  |  | Depressive Disorder |  | 76 (2.24%) | 87 (2.56%) | 0.87 [0.64-1.19] | 0.383 |
| English Primary Language | Spanish Primary Language | Anxiety and/or Depressive Disorder | 2,591 | 114 (4.40%) | 77 (2.97%) | **1.50 [1.20-2.02]** | **0.006** |
|  |  | Anxiety Disorder |  | 73 (2.83%) | 56 (2.16%) | 1.31 [0.92-1.87] | 0.130 |
|  |  | Depressive Disorder |  | 68 (2.62%) | 38 (1.47%) | **1.81 [1.21-2.70]** | **0.003** |

^a^Anxiety disorders included ICD-10-CM codes F41 and F43, while depressive disorders included ICD-10-CM codes F32 and F33

^b^Odds ratios were calculated by directly diving the odds of the event in one group by the odds of the same event in the comparison group after propensity score matching for age, sex, race, ethnicity, English primary language, history of chemotherapy, history of radiation, tumor summary stage, and history of stroke, heart failure, or myocardial infarction. Race, ethnicity, and English primary language were removed from propensity score matching as necessary for comparisons.

^c^Any minority refers to any individual of a minority race in the United States (any non-Caucasian race), including those identifying as black or African American, Asian, other races, American Indian or Alaska Native, or Native Hawaiian or Other Pacific Islander.

**Supplemental Table 4.** Odds ratios comparing likelihood of receiving pharmacotherapy or psychotherapy after selected anxiety and/or depressive disorder within 2 years after HNC diagnosis based on demographic characteristics, after propensity matching: sensitivity analysis omitting participants with missing demographic data^a^

| **Group 1** | **Group 2** | **Treatment Type** | **n per group after propensity score matching** | **n (%) Treated, Group 1** | **n (%) Treated, Group 2** | **OR [95% CI]** | **p-value** |
| --- | --- | --- | --- | --- | --- | --- | --- |
| Male | Female | Pharm- acotherapy | 1,284 | 594 (46.26%) | 685 (53.25%) | **0.75 [0.65-0.88]** | **<0.001** |
|  |  | Psych- otherapy |  | 51 (3.97%) | 57 (4.44%) | 0.89 [0.61-1.31] | 0.555 |
| White/ Caucasian | Any minority^c^ | Pharm- acotherapy | 541 | 276 (51.02%) | 233 (43.07%) | **1.38 [1.08-1.75]** | **0.009** |
|  |  | Psych- otherapy |  | 25 (4.62%) | 24 (4.44%) | 1.04 [0.59-1.85] | 0.884 |
| Non- Hispanic/ Latino | Hispanic/ Latino | Pharm- acotherapy | 164 | 96 (58.54%) | 74 (45.12%) | **1.72 [1.11-2.66]** | **0.015** |
|  |  | Psych- otherapy |  | 13 (7.93%) | 11 (6.71%) | 1.20 [0.52-2.76] | 0.672 |
| English Primary Language | Spanish Primary Language | Pharm- acotherapy | 81 | 40 (49.38%) | 20 (24.69%) | **2.98 [1.53-5.80]** | **0.001** |
|  |  | Psych- otherapy |  | ≤10 (≤12.35%) | ≤10 (≤12.35%) | NA^b^ | NA^b^ |

^a^Odds ratios were calculated by directly diving the odds of the event in one group by the odds of the same event in the comparison group after propensity score matching for age, sex, race, ethnicity, English primary language, history of chemotherapy, history of radiation, tumor summary stage, and history of stroke, heart failure, or myocardial infarction. Race, ethnicity, and English primary language were removed from propensity score matching as necessary for comparisons.

^b^TriNetX does not show n less than 10, so associated odds ratio calculations were omitted in these cases.

^c^Any minority refers to any individual of a minority race in the United States (any non-Caucasian race), including those identifying as black or African American, Asian, other races, American Indian or Alaska Native, or Native Hawaiian or Other Pacific Islander.
